# Large Scale Screening of Ethnomedicinal Plants for Identification of Potential Antibacterial Compounds

**DOI:** 10.3390/molecules21030293

**Published:** 2016-03-14

**Authors:** Sujogya Kumar Panda, Yugal Kishore Mohanta, Laxmipriya Padhi, Young-Hwan Park, Tapan Kumar Mohanta, Hanhong Bae

**Affiliations:** 1 Department of Zoology, North Orissa University, Baripada, Odisha 757003, India; sujogyapanda@gmail.com (S.K.P.); omsai.manasi@gmail.com (L.P.); 2 Department of Botany, North Orissa University, Baripada, Odisha 757003, India; ykmohanta@gmail.com; 3 School of Biotechnology, Yeungnam University, Gyeongsan 712749, Korea; pyhasdf@nate.com; 4 Free Major of Natural Sciences, College of Basic Studies, Yeungnam University, Gyeongsan 712749, Korea

**Keywords:** multiple antibiotic resistances, human pathogens, antibacterial activity, medicinal plants

## Abstract

The global burden of bacterial infections is very high and has been exacerbated by increasing resistance to multiple antibiotics. Antibiotic resistance leads to failed treatment of infections, which can ultimately lead to death. To overcome antibiotic resistance, it is necessary to identify new antibacterial agents. In this study, a total of 662 plant extracts (diverse parts) from 222 plant species (82 families, 177 genera) were screened for antibacterial activity using the agar cup plate method. The aqueous and methanolic extracts were prepared from diverse plant parts and screened against eight bacterial (two Gram-positive and six Gram-negative) species, most of which are involved in common infections with multiple antibiotic resistance. The methanolic extracts of several plants were shown to have zones of inhibition ≥ 12 mm against both Gram-positive and Gram-negative bacteria. The minimum inhibitory concentration was calculated only with methanolic extracts of selected plants, those showed zone of inhibition ≥ 12 mm against both Gram-positive and Gram-negative bacteria. Several extracts had minimum inhibitory concentration ≤ 1 mg/mL. Specifically *Adhatoda vasica*, *Ageratum conyzoides*, *Alangium salvifolium*, *Alpinia galanga*, *Andrographis paniculata*, *Anogeissus latifolia*, *Annona squamosa*, *A. reticulate*, *Azadirachta indica, Buchanania lanzan, Cassia fistula, Celastrus paniculatus, Centella asiatica*, *Clausena excavate*, *Cleome viscosa*, *Cleistanthus collinus*, *Clerodendrum indicum*, *Croton roxburghii*, *Diospyros melanoxylon*, *Eleutherine bulbosa*, *Erycibe paniculata*, *Eryngium foetidum*, *Garcinia cowa*, *Helicteres isora, Hemidesmus indicus, Holarrhena antidysenterica, Lannea coromandelica, Millettia extensa*, *Mimusops elengi, Nyctanthes arbor-tristis*, *Oroxylum indicum*, *Paederia foetida*, *Pterospermum acerifolium*, *Punica granatum*, *Semecarpus anacardium*, *Spondias pinnata*, *Terminalia alata* and *Vitex negundo* were shown to have significant antimicrobial activity. The species listed here were shown to have anti-infective activity against both Gram-positive and Gram-negative bacteria. These results may serve as a guide for selecting plant species that could yield the highest probability of finding promising compounds responsible for the antibacterial activities against a broad spectrum of bacterial species. Further investigation of the phytochemicals from these plants will help to identify the lead compounds for drug discovery.

## 1. Introduction

Medicinal plants have long been used to treat diseases [1,2]. Plants are commonly used as sources of new pharmaceuticals due to the presence of promising therapeutic compounds. Infectious diseases play a significant role in the deaths of millions of people worldwide, in part due to the mutagenic nature of the bacterial genome. Moreover, the exchange and uptake of plasmids among bacteria results in the development of multiple antibiotic resistant strains. Antimicrobials from different plants have enormous therapeutic potential and lesser side effects than synthetic antibiotics [3,4]. Accordingly, it is desirable and essential to develop an effective, safe and natural product to control multiple drug resistance (MDR) pathogens. Medicinal plants contain active principles generated by various natural metabolic processes and each plant species has its own metabolome that governs the presence of chemical components or bioactive molecules [5].

India is one of the richest countries in the world with regards to the genetic resource of medicinal plants [6]. The country has a wide range of topography and climate, which influences its vegetation and floristic composition. Worldwide searches for antimicrobial agents continued to focus on lower plants, fungi and bacteria [7]. There are many approaches that can be used to select plants of potential therapeutic interest [8]. Compounds can be identified through random, ethno- (including ethnobotanical, ethnomedical and ethnopharmacological) and ecological searches [9]. The random collection of plant samples from certain habitats with high species diversity (for example tropical rain forests) can be very useful for identification of novel chemical entities. However, this method is time consuming and labor intensive [10]. This kind of sampling is most likely to be used in industry to evaluate the industrial approach and most likely to be used for evaluating plants for bioactive compounds [9].

Several studies have provided evidence that the antimicrobial compounds isolated from different solvent extracts never provided the expected final output based on the activity of crude extracts and fractions [11,12]. This is probably because different plant metabolites often work in combination with other compounds to regulate microbial infections and may therefore not be effective alone [13]. For these reasons, we investigated a large number of plant species that have not yet been examined for their antimicrobial activities. The solvent (extraction agent) used to prepare phytopharmaceuticals must be able to dissolve all key phytoconstituents, which should be nontoxic and easy to remove through excretion. Traditional healers typically use aqueous extracts. The activity of effective aqueous extracts used by traditional healers is based on indirect effects that work by stimulating the immune system of the host rather than killing the pathogens [12]. Therefore, in the present study, an aqueous extract was used in the preliminary screening (agar diffusion method). It is believed that methanol could efficiently penetrate the cell membranes, permitting the extraction of high amounts of endocellular components in contrast to low polarity solvents such as chloroform and petroleum ether which can only extract extracellular material. Methanol primarily dissolves polar constituents together with medium and low polarity compounds extracted by cosolubilization. Therefore, the present investigation was conducted to evaluate both the aqueous and methanolic (80%) extracts of different plants belonging to a wide range of families based on random sampling. The result presented herein will be useful to further search of novel plants with antibacterial properties.

## 2. Results and Discussion

A total of 222 plant species (177 genera) collected from Mayurbhanj, Odisha, India were screened using the agar cup plate method. Screened samples were selected based on random screening and ethno medicinal uses [14]. Eight species of bacteria (two Gram-positive and six Gram-negative), mostly involved in common infections such as gastroenteritis, diarrhea, dysentery, skin diseases, and food and water contamination, were used to screen for antimicrobial activity. Two different solvents: methanol (80%) and water were used to prepare the crude extracts of different species for screening (Table 1).

The zones of inhibition shown by each plant are listed in Table 2. In total, 258 parts belonging to 222 species, 177 genus and 83 families (258 methanol extracts + 258 aqueous extracts) were tested for antibacterial properties. Of them, 125 leaf extracts, 19 bark extracts, eight whole plant extracts, four stem extracts, four root extracts, three fruit extracts, three rhizome extracts and one bulb part showed anti-bacterial activity. A total of 165 methanol extracts were found to be active against the tested strains (at least one or more bacterial strain) while the results with aqueous extracts were comparatively fewer (127).

About 146 methanol extracts showed antibacterial activity against Gram-positive (56.58%) bacteria, while 137 extracts were active against Gram-negative bacteria (53.10%) (Table 3). Similarly 89 aqueous extracts showed antibacterial activity against Gram-positive (34.49%) species followed by 102 extracts against Gram-negative bacteria (39.53%). Among them, 10 methanol extract samples were strongly inhibitory of the tested bacteria (zones of inhibition ≥ 20 mm). A total of 34 methanol extracts were moderately inhibitory to the test bacteria (zones of inhibition in between 15–20 mm) and 160 methanol extracts were weakly inhibitory (zone of inhibition < 15 mm) in comparison to the standard antibiotics gentamycin and ciprofloxacin (Table 3).

Aqueous extracts have commonly been used to test for antibiotic activity, especially in preliminary studies [15]. It is believed however that alcoholic solvents can efficiently penetrate cell membranes, permitting extraction of higher levels of endo-cellular components than solvents with lower polarity such as chloroform and petroleum ether [16]. In this way, alcohol dissolves primarily polar constituents together with medium and low polar compounds extracted by cosolubilization [17]. The antibacterial activities of methanolic extracts were found to be more potent than those of aqueous extracts. Gram-positive bacteria are already known to be more susceptible to plant extracts than Gram-negative bacteria [18,19]. These differences may be attributed to the fact that the cell wall in Gram-positive bacteria is single layered, whereas that of Gram-negative cells is multilayered [18,19]. Alternatively, the passage of the active compound through the Gram-negative cell wall may be inhibited due to rupture of ion channels. However, numerous plant extracts showed inhibition against Gram-negative bacteria. This is also in agreement with the results of Nikaido [20], who reported that Gram-negative bacteria have a hydrophilic membrane because of the presence of lipopolysaccharides. Thus, a small hydrophilic molecule can pass through the outer membrane. Conversely, this outer membrane also allows passage of lipophilic compounds and macromolecules. Understanding the permeation properties of the outer membrane of the microorganisms is prerequisite to know about the antibacterial activity of a solute. Thus, since the methanol extracts used in this study are partially soluble in water, they penetrate the outer membrane of Gram-negative bacteria and disturb the inside of the cell hampering cellular function and metabolism causing loss of cellular constituents, and eventually leading to cell death. Similar results have been reported in other studies as well [21,22].

Some of the important plant families that exhibited antimicrobial activities were Acanthaceae (four), Anacardiaceae (five), Apocyanaceae (four), Asteraceae (six), Ceasalpiniaceae (four), Combretaceae (seven), Ebenaceae (four), Euphorbiaceae (six), Fabaceae (eight), Myrataceae (four), Rubiaceae (four), Rutaceae (four), and Verbenaceae (four).

In total, 90 plants species (82 genera from 39 families) were unable to inhibit the tested pathogens. However, among these 25 families representing other species were active against the test pathogens, so in total plants from 15 families did not show antibacterial activity, namely *Barleria strigosa* Willd., *Hygrophila auriculata* (K. Schum.) Heine, (Lf, Acantahceae); *Agave sisalana* Perr. ex Engl. (Lf, Agavaceae), *Amaranthus spinosus* L. (Lf, Amaranthaceae), *Thevetia peruviana* (Pers.) K. Schum. (Lf, Apocynaceae); *Rauvolfia tetraphyla* (L.) Benth. (Lf, Sd, Apocynaceae); *Adenostemma lavenia* (L.) Kuntze, *Eclipta prostrata* (L.), *Sphaeranthus indicus* L., *Stereospermum chelonoides* (L.f.) DC. (Lf, Asteraceae); *Bixa orellana* L. (Lf, Bixaceae); *Bauhinia malabarica* Roxb., *B. purpurea* L., *B. roxhurghiana* Voigt, *Caesalpinia pulcherrima* (L.) Sw., *Saraca asoca* (Roxb.) de Wilde (Lf, Caesalpiniaceae); *Chenopodium album* L. (Wp, Chenopodiaceae); *Commelina suffruticosa* Blume, *Cyanotis tuberosa* (Roxb.) Schult & Schult.f., *Floscopa scandens* Lour. (Lf, Commelinaceae); *Argyreia nervosa* (Burm. f.) Boj., *A. speciosa* (Burm. f.) Boj., *Merrimia umbellate* (L.) Hall. f., *Operculina turpethum* (L.) Silvo-Mano (Lf, Convolvulaceae), *Ipomoea nil* (L.) Roth. (Rt, Convolvulaceae); *Cucumis sativus* L., *Cucurbita maxima* Duch. ex Lam., *Lagenaria siceraria* (Molina) Standley, *Luffa acutangula* (L.) Roxb., *Momordica dioica* Roxb. ex Willd., *Solena heterophylla* Lour. (Lf, Cucurbitaceae); *Dioscorea pentaphylla* L. (Rh, Dioscoreaceae); *Drosera burmannii* Vahl., *Drosera indica* L. (Lf, Droseraceae), *Euphorbia nivulia* Buch.-Ham., *Sebastiania chamaelea* (L.) Muell. Arg., *Trewia nudiflora* L. (Lf, Euphorbiaceae); *Flacourtia ramontchi* L. Herit. (Lf, Flacourtiaceae), *Atylosia scarabaeoides* (L.) Benth., *Butea monosperma* (Lam.) Taub., *Crotalaria albida* Heyne ex Roth., *Crotalaria prostrata* Rottl. ex Willd., *Dalbergia lanceolaria* L.f., *Dalbergia pinnata* (Lour.) Prain, *Flemingia chappar* Buch.-Ham.ex Benth., *Indigofera prostrate* Willd., *Lablab purpureus* (L.) Sweet, *Mucuna pruriens* (L.) DC., *Pueraria tuberose* (Roxb. ex Willd.) DC., *Sesbania bispinosa* (Jacq.) W.F. Wight, *Teramnus labialis* (L.f.) Spreng., *Uraria rufescens* (DC.) Schindl. (Lf, Fabaceae); *Derris indica* (Lam.) Bennet (Sd, Fabaceae), *Flemingia strobilifera* (L.) R.Br. (Rt, Fabaceae); *Exacum bicolor* Roxb. (Lf, Gentianaceae); *Vallisneria natans* (Lour.) Hara (Hydrocharitaceae); *Hypericum japonicum* Thunb. Ex. Murray (Lf, Hypericaceae), *Curculigo orchioides* Gaertn. (Rt, Hypoxidaceae); *Litsea monopetala* Roxb. (Bk, Lauraceae); *Utricularia bifida* L. (Lf, Lentibulariaceae); *Asparagus racemosus* Willd., *Iphigenia indica* (L.) A Gray ex Kunth (Rt, Liliaceae); *Ammannia baccifera* L., *Lawsonia inermis* L. (Lf, Lythraceae); *Hibiscus furcatus* Willd., (Lf, Malvaceae); *Mimosa pudica* L., *Xylia xylocarpa* (Roxb.) Taub. (Lf, Mimosaceae); *Artocarpus heterophyllus* Lam., *Ficus benghalensis* L., *F. religiosa* L. (Lf, Moraceae), *Musa paradisiaca* L. (St, Musaceae); *Embelia tsjeriam-cottam* A. DC. (Lf, Myrsinaceae); *Boerhavia diffusa* L. (Lf, Nyctaginaceae); *Jasminum arborescens* Roxb., (Lf, Olacaceae); *Oxalis corniculata* L. (Wp, Oxalidaceae); *Cymbopogon flexuosus* (Nees ex Steud.) Wats., *Cynodon dactylon* (L.) Pers., (Wp, Poaceae); *Ziziphus rugosa* Lam. (Lf, Rhamnaceae); *Gardenia gummifera* Lf, *Haldinia cordifolia* (Roxb.) Rids, *Rubia cordifolia* L. (Lf, Rubiaceae); *Litchi chinensis* Sonner (Lf, Sapotaceae), *Solanum nigrun* L., *S. erianthum* D. Don (Lf, Solanaceae); *Symplocos racemosa* Roxb. (Lf, Symplocaceae); *Trapa natens* L. (Lf, Trapaceae); *Callicarpa macrophylla* Vahl, *Tectona grandis* Lf (Lf, Verbenaceae), *Costus speciosus* (Koenig) Sm. and *Curcuma amada* Roxb.(Lf, Zingiberaceae). The methanol extracts from the diverse parts of selected plants that showed zones of inhibition greater than 12 mm against both Gram-positive and Gram-negative bacteria were further tested to determine the corresponding MIC values.

**Figure 1:**
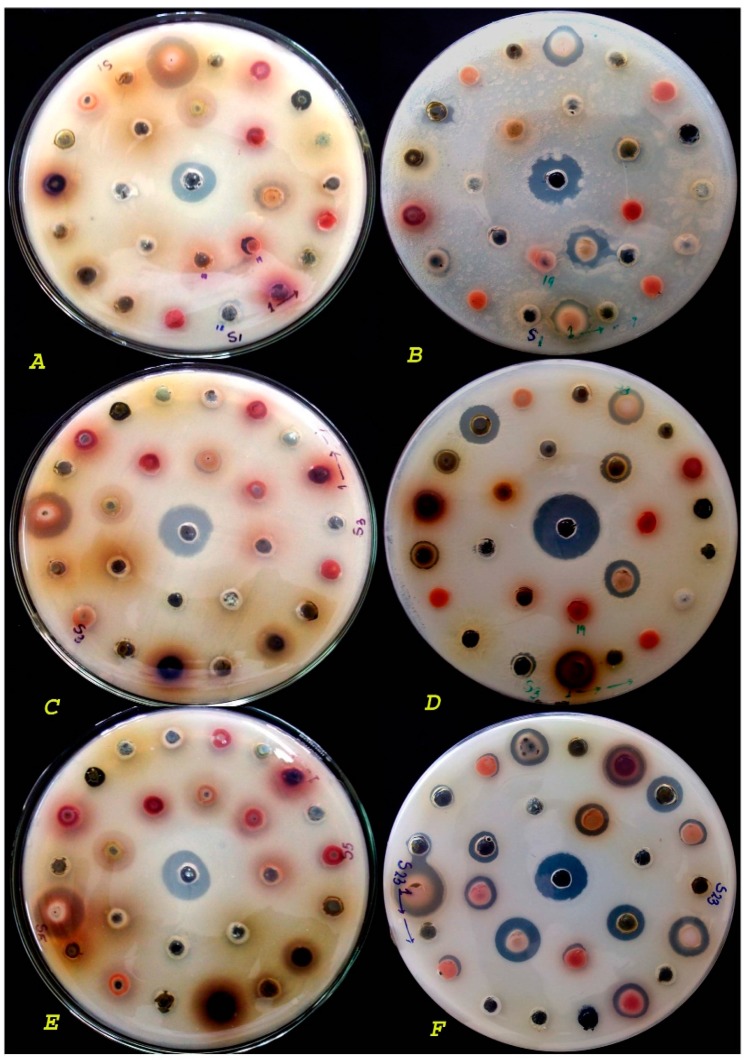
Screening of plant extracts; (**A**) Plant extracts (methanol) against *E. coli*; (**B**) Plant extracts (water) against *E. coli*; (**C**) Plant extracts (methanol) against *S. aureus*; (**D**) Plant extracts (water) against *S. aureus*; (**E**) Plant extracts (methanol) against *S. typhimurium*; (**F**) Plant extracts (methanol) against *V. cholera*.

The broth dilution technique determines the antimicrobial activities measured as MICs (Figure 1). Four different bacteria *viz*. *S. aureus*, *B. cereus*, *S. flexneri* and *V. cholerae* were tested for this and results are reported in Table 4 (Figure 1). The calculated MIC of the majority of the strains was between 62–4000 µg/mL. In total, 65 extracts were tested with four bacteria (65 × 4 = 260), of which 79 hits exhibited MIC ≤ 500 µg/mL. The results in Table 4 indicate that most of the test strains show inhibition zones at a concentration ≤ 2000 µg/mL, while half of the extracts were active with a MIC ≤ 1000 µg/mL (Figure 1). MIC values lower than 250 µg/mL were also obtained for quite a few extracts. The lowest MIC value for *B. lanzan* (bark), *C. fistula* (leaf), *N. arbortristis* (bark), *E. bulbosa* (bulb) was obtained against *S. aureus* (MIC < 200 µg/mL). However, *E. bulbosa* (bulb) demonstrated the lowest MIC among all four test bacteria (22–125 µg/mL).

Unlike the agar cup method, the broth dilution results also shown that Gram-negative bacteria (*S. flexneri* and *V. cholerae*) are more resistant than Gram-positive (*B. cereus* and *S. aureus*) ones to the majority of extracts. Furthermore, it was observed that a few of the extracts are insensitive in the broth dilution method with MIC ≥ 5000 µg/mL, although they displayed inhibition zones in the agar cup method.

Ahmad *et al.* [23] and Valasraj *et al.* [24] tested 82 and 78 Indian medicinal plants, respectively, against several pathogenic and opportunistic microorganisms. Perumalsamy and Ignacimuthu [25] screened a series of 30 Indian medicinal plants using the disc diffusion method against both Gram-positive and Gram-negative bacteria. Srinivasan *et al.* [26] tested 50 medicinal plants belonging to 26 families for antimicrobial activity. Ahmad and Beg [27] also examined 45 Indian medicinal plants against different drug resistant bacteria and yeast. Ram *et al.* [28] screened the antimicrobial properties of 23 medicinal plants from Eastern Ghats, India against three bacterial species and one fungal species.

Kumar *et al.* [29] investigated a series of Indian medicinal plants against several bacteria and fungi. Parekh and Chanda [30] screened the antibacterial activity of aqueous and alcoholic extracts of 34 medicinal plants, belonging to 28 families against six bacteria from Enterobacteriaceae by agar well diffusion method. In all of these studies the ethanol and methanol extracts were more active than aqueous extracts for all tested plants. Antibacterial activity of alcoholic extracts of 15 Indian medicinal plants, against ESβL-producing multidrug resistant bacteria was studied by Ahmad and Aqil [31]. All these finding are in accordance with the results obtained in our experiments.

This study led to identification of plants from northern Odisha with antimicrobial activities against common pathogens. Some of the active species have already been shown to have similar activity. Additionally, the effects of some of these plants *viz*. *Justicia adhatoda*, *Alangium salvifolium*, *Achyranthes aspera*, *Andrographis paniculata*, *Aristolochia indica*, *Azadirachta indica*, *Calotropis procera*, *Cassia fistula*, *Cassia occidentalis*, *Cassia tora*, *Carica papaya*, *Cleistanthus collinus*, *Croton roxburghii*, *Cleome viscosa*, *Hemidesmus indicus*, *Holarrhena antidysenterica*, *Leea indica*, *Pergularia demia*, *Moringa oleafera*, *Punica granatum*, *Sida acuta*, *Semecarpus anacardium, Spondias pinnata, Tamarindus indica*, and *Vitex negundo*, were previously described by our group and other researchers [14,15,17,23,24,25,26,27,28,29,31]. Plants for which antibacterial activity is reported here for the first time include: *Alpinia galanga*, *Vernonia squarrosa*, *Euonymus glaber*, *Garcinia cowa*, *Commelina paludosa*, *Erycibe paniculata*, *Indigofera cassoides*, *Millettia extensa*, *Pterocarpus marsupium*, *Tephrosia purpurea*, *Desmodium gangeticum*, *Acacia leucophloea*, *Ardisia solanacea*, *Eucalyptus citriodora*, *Ixora pavetta*, *Mitragyna parvifolia*, *Wendlandia tinctoria*, *Acronychia pedunculata*, *Scoparia dulcis*, *Solanum virginianum*, *Grewia elastica*, *Dalbergia volubilis*, *Litsea glutinosa*, *Antidesma ghaesembilla*, *Opuntia vulgaris* and *Biophytum reinwardti.*

In the present study, high degrees of differences in susceptibility among dissimilar bacteria were observed. Typically each plant is different due to its unique phytoconstituents. While some are safe and effective for specific uses, others may not be. It is commonly believed that medicinal plants/drugs are safe and free from the side effects, however, this is not true for every case. Several medicinal plants can produce undesirable side effects and can even be very toxic [32]. A specific plant part may have various constituents and other parts may be toxic. To verify the biological activity and toxicity of medicinal plants, a basic screening step is very necessary for preliminary safety evaluation of plant extracts/compounds prior to further development and commercialization. Ideally, a cell line cytotoxicity study can rule out false positive bioactivity ensuing from a general toxic effect of the plant extract(s). As in the present study, we screened a large numbers of plants with different bacteria, we lack this toxicity study. On the other hand, many of our tested plants are used as ethnomedicine and their safety and efficacy are already reported. Nevertheless, more of the compounds should be subjected to animal and human studies to determine their effectiveness in whole organism systems, including in particular toxicity studies as well as an examination of their effects on beneficial microbiota [33].

## 3. Experimental Section

### 3.1. Study Area

**Figure 2:**
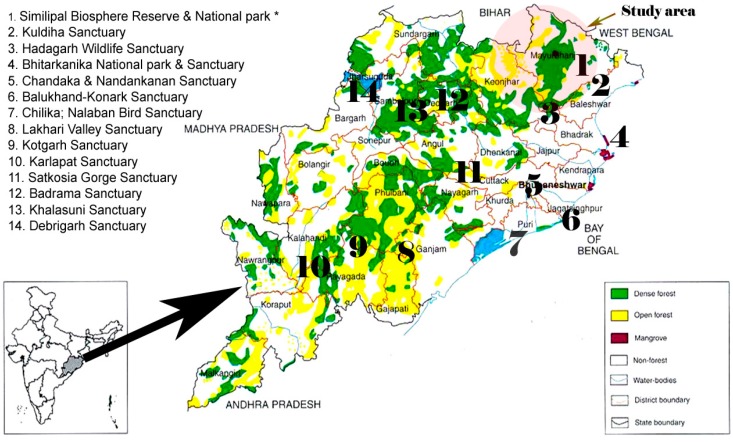
Forest areas of the state of Odisha showing sampling sites and biodiversity spots.

The northern part of Orissa offers unique opportunities to study plants used by indigenous populations. About 62 ethnic tribal communities have been reported in the study area most of which inhabit the forest. These communities meet all of their needs including food and primary healthcare, from forest resources. Of 62 tribal communities, 30 (48%) and several aboriginals are found in the district of Mayurbhanj (the largest district of Odisha; area, 10,418 sq km; forest cover, 4392 sq. km; population, 2,513,895 based on a 2011 census) and Keonjhar (area, 8240 sq km; forest cover, 2525 sq. km; population, 18,017,733/2011 census). The Similipal Biosphere Reserve (SBR, 5569 sq. km) is located in the heart of the Mayurbhanj district, adjoining the Keonjhar district, and its rich biodiversity is known internationally (Figure 2). Both districts offer unique opportunities to study indigenous medicinal plants used by populations. The major local tribes live in this region includes Santal, Kolha, Bathudi, Bhumij, Munda and Gond are the major tribes whereas the Mankidia, Lodha, Kisan and Baiga are the minor tribal groups that inhabit the area. The Santal constitutes the largest tribal race and are scattered throughout the regions. The social, cultural and religious life of aboriginal people is influenced by the nature and natural resources available in and around their habitat which provide the food, medicine, shelter, and various other materials and cultural needs. Both districts are largely covered with forest containing different climatic zones and a wide range of vegetation. It is estimated that more than 2000 plant species are available from both districts; however it is not practical to screen all of them. To reduce the large species range, the study was focused only on medicinal herbs. We sampled mostly leaf materials (unless ethnomedicinal information was available regarding other parts), because leaves are a renewable resource and it is also easier to recollect leaves from the same plant for follow-up work. The identification and voucher specimen deposition of these medicinal plants was conducted at the Post Graduate Department of Botany, North Orissa University (Baripada, Odisha, India).

### 3.2. Processing

The bark, flowers, fruits, leaves, roots, seeds, aerial shoots and stems of plants were collected separately during field trips to different places in the Similipal Biosphere Reserve. The roots were dug out from the soil and the adhering soils were removed by shaking and washing. Healthy leaves were plucked from large plants and washed with sterile distilled water. Following collection, the healthy leaves were dried at low temperature without allowing the growth of any type of fungi, or bacteria. The dried leaves, roots and stems were powdered separately using a mortar and pestle then passed through a 40–60 mm mesh size sieve to obtain uniform powdered samples.

#### Preparation of Plant Extracts

A total of 100 g of each powdered sample was dissolved in 200 mL of sterile distilled water and 80% methanol separately in wide mouth bottles. The aqueous samples were then steamed with distilled water for 30 minutes, after which they were stored overnight. Next, the suspensions were filtered separately (Whatman No. 40 paper) and used to investigate the antimicrobial properties. The methanol extracts were dried in a rotary evaporator at 50 °C and stored in a refrigerator until further analysis.

### 3.3. Antibacterial Activity

#### 3.3.1. Test Bacterial Strains

The antibacterial activity was tested against the strains *Bacillus cereus* (medical isolate), *Staphylococcus aureus* MTCC 1144, *Escherichia coli* MTCC 1098, *Salmonella typhimurium* MTCC 3216, *Shigella sonnei*, *Shigella dysentriae*, *Shigella flexneri* (medical isolates) and *Vibrio cholerae* MTCC 3904.

#### 3.3.2. Maintenance of Bacteria

Bacterial cultures were maintained on nutrient agar (NA) slants at 4 °C. Bacterial species were activated by streaking culture from the slants onto Muller Hinton Agar (MHA) plates and then incubating overnight at 37 °C. Individual colonies were selected from each plate and transferred to nutrient broth, after which they were incubated for 1 day at 37 °C prior to the tests.

### 3.4. Antibiotics

Different antibiotics (Hi Media Pvt. Ltd., Mumbai, India) at the given concentrations were used to determine the antibiotic sensitivity profile of the reference bacteria including Amikacin (Ak) 30 μg; Amoxicillin, (Aug) 10 μg; Ampicillin (A) 10 μg; Cefoxitin (Ctn) 10 μg; Ceftriaxone (Cez) 10 μg; Cephotaxime (Ce) 30 μg; Chloroamphinecol (Ch) 10 μg; Ciprofloxacin (C) 10 μg; Erythromycin (E) 15 μg; Gatifloxacin (Gf) 30 μg; Gentamicin (G) 10 μg; Levofloxacin (Lvx) 5 μg; Naladixic acid (Nal) 30 μg; Ofloxacin (Ofl) 5 μg; Polymyxin-B (Pb) 300 unit; Streptomycin (St) 10 μg; Tetracycline (Te) 10 μg and Vancomycin (Vn) 30 μg.

### 3.5. Sensitivity Tests

An antibiogram with commonly used antibiotics was conducted by the disc diffusion method [34,35]. The antibiotic sensitivity was tested in MHA plates (Himedia Laboratories, Mumbai, India). The test microbes were removed from the slants aseptically with inoculating loops and transferred to separate test tubes containing 5.0 ml of sterile distilled water. The inocula were added until the turbidity was 0.5 McFarland (10^8^ CFU°). For each bacterial species, 1 mL of the test tube suspension was added to 15–20 mL of nutrient agar and transferred to an agar plate (90 mm diameter). After cooling the inoculated agar at room temperature for 25 min, the antibiotic sensitivity test discs were placed on the surface of the solid agar. The plates were incubated at 37 °C and then examined for zones of inhibition. The results are summarized in Table 5 below.

### 3.6. Agar Cup Method

The agar cup method was used to investigate the antibacterial activity of the extracts [14]. Overnight Muller Hinton Broth cultures of the test organisms were seeded onto MHA plates after which wells approximately 6 mm in diameter and 2.5 mm deep were made on the surface of the solid medium using a sterile borer. The plates were then turned upside down and the wells were labeled with a marker. Each well was subsequently filled with 50 µL of test sample. Sterile 80% methanol was used as negative control, while gentamicin and ciprofloxacin were used as positive controls. The plates were incubated at 37 °C for 24 h after which the plates were removed and zones of inhibition were measured using the Hi Media antibiotic scale and the results were tabulated. Extracts with zones of inhibition greater than or equal to 8 mm diameter were considered as positive.

### 3.7. Minimum Inhibitory Concentration (MIC)

To determine the MIC, a microdilution technique was adopted using 96-well microtiter plates and tetrazolium salt, 2,3,5-triphenyltetrazolium chloride (TTC) as per the previous report [14]. The microplates were sealed and incubated at 37 °C at 130 rpm and observed for growth of the microorganisms.

## 4. Conclusions

The present study provides informative data regarding plants which have never been studied previously for the presence of antimicrobial activity against pathogenic bacteria. Further study is required to identify the active compounds, synergetic effects, toxicity, and safety of these plants and eventually clinical evaluations.

## Figures and Tables

**Table 1:** Summary of antibacterial activity among the test plants.

Scrutiny	No. of Extracts Reported as Antibacterial (%)		
	Element	Methanol Extract	Aqueous Extract
Total number of plant species tested—22	Gram positive	146 (56.58%)	89 (34.49%)
Total number of Genus tested—177	Gram negative	137 (53.10%)	102 (39.53%)
Total number of family tested—83	*B. cereus*	108 (41.86%)	50 (19.37%)
Total number of parts tested = 258	*S. aureus*	124 (48.06%)	76 (29.45%)
Leaves-125; Bark-19; Whole part-08; Stem-04	*E. coli*	68 (26.35%)	45 (17.44%)
Root-04; Rhizome-03; Fruit-03 and Bulb-01	*S. typhimurium*	65 (25.19%)	41 (15.89%)
Total number of methanol extracts active—165	*S. dysentriae*	50 (19.37%)	22 (8.52%)
Total number of aqueous extracts active—127	*S. flexneri*	66 (25.58%)	28 (10.85%)
Number of species do not show activity—90 species	*S. sonnei*	47 (18.21%)	24 (9.30%)
Number of extracts do not show activity	*V. cholerae*	72 (27.90%)	38 (14.72%)
(93 methanol + 131 aqueous = 224)	Zone ≥ 20 mm	10 (3.87%)	0
Total number of family show activity—68	Zone 15–20 mm	34 (13.17%)	9 (3.48%)
Total number of family do not show activity—15	Zone < 15	160 (62.01%)	121 (46.89%)

**Table 2:** Results of screening of plants from Northern Odisha, India.

Plant Description	Zone of Inhibition in mm									
	PU	E	Bc	Sa	Ec	St	Sd	Sf	Ss	Vc
**Acanthaceae**										
*Andrographis paniculata* (Burm. f.) Nees	Lf	A	14	12	11	10	12	-	14	-
		M	12	12	14	13	-	12	16	-
	St	A	12	12	12	12	-	-	-	-
		M	12	14	16	-	10	14	15	10
*Barleria cristata* L.	Lf	A	12	12	-	-	-	-	-	-
		M	14	18	-	-	-	-	-	-
*Adhatoda vasica* Nees	Lf	A	11	10	-	12	12	-	12	11
		M	14	12	10	10	10	10	12	10
**Acoraceae**										
*Acorus calamus* L.	Rh	A	-	-	-	-	-	-	-	09
		M	12	18	-	-	-	10	-	12
**Alangiaceae**										
*Alangium salvifolium* (C.B.Clarke) W.W.Sm. & Cave	Lf	A	12	10	10	-	-	-	-	-
		M	14	16	12	12	12	12	-	-
*Alpinia galangal* (Linn.) Wild.	Lf	A	-	-	-	-	-	-	-	-
		M	14	12	10	10	-	16	-	14
**Amaranthaceae**										
*Achyranthes aspera* L.	Wp	A	-	-	-	11	-	-	-	09
		M	14	12	12	12	-	-	-	08
*Achyranthes bidentata* L. Blume	Wp	A	-	-	-	-	-	-	-	-
		M	12	12	-	-	-	-	-	-
*Cyathula prostrata* L. Blume	Lf	A	-	-	-	-	-	-	-	-
		M	-	-	-	-	-	-	-	10
**Anacardiaceae**										
*Buchanania lanzan* Spreng	Bk	A	15	12	-	-	-	-	-	-
		M	16	14	-	13	-	12	14	10
*Lannea coromandelica* (Houtt.) Merr.	Bk	A	12	12	-	09	10	-	-	-
		M	-	12	-	14	10	10	-	10
*Mangifera indica* L.	Lf	A	-	-	-	-	-	-	-	-
		M	10	14	-	-	-	-	-	-
*Semecarpus anacardium* L.f.	Fr	A	11	14	-	12	-	-	-	-
		M	12	15	-	13	-	14	-	11
*Spondias pinnata* (L.f.) Kurz	Lf	A	-	-	10	-	-	-	-	-
		M	10	14	11	12	-	14	-	13
**Annonaceae**										
*Annona reticulata* L.	Lf	A	-	-	-	-	-	12	-	12
		M	12	12	-	-	12	13	-	12
*Annona squamosa* L.	Lf	A	-	12	-	-	-	-	12	-
		M	13	16	-	-	12	-	14	12
**Apiaceae**										
*Centella asiatica* (L.) Urb.	Wp	A	12	12	10	-	-	-	-	10
		M	13	14	10	-	12	-	12	14
*Eryngium foetidum* L.	Lf	A	09	12	12	-	13	-	11	-
		M	10	14	13	-	13	-	12	-
**	St	A	11	13	12	-	-	-	09	-
		M	12	18	14	-	12	12	11	10
**Apocyanaceae**										
*Alstonia scholaris* (L.) R.Br.	Lf	A	-	-	-	-	-	-	-	-
		M	14	11	-	-	-	10	-	12
*Alstonia venenata* R.Br.	Lf	A	-	-	-	-	-	-	-	-
		M	12	-	-	-	-	14	-	10
*Holarrhena antidysenterica* Wall ex. A.DC.	Lf	A	18	12	12	14	-	-	11	-
		M	15	12	12	14	-	12	12	12
*Ichnocarpus frutescens* (L.) W.T.Aiton	Lf	A	-	-	-	-	-	-	-	-
		M	12	11	-	-	-	-	12	-
*Rauvolfia serpentina* (L.) Benth. ex Kurz	Rt	A	-	-	-	-	-	-	-	-
		M	-	-	10	-	-	-	12	-
**Araceae**										
*Acorus calamus* L.	Rh	A	-	-	09	-	12	-	-	09
		M	-	-	12	12	14	-	-	12
**Aristolochiaceae**										
*Aristolochia indica* L.	Lf	A	-	12	-	-	-	-	-	-
		M	12	10	-	-	10	10	-	-
**Asclepiadaceae**										
*Calotropis procera* (Aiton) Dryand.	Lt	A	-	12	-	-	-	-	-	12
		M	-	14	-	-	-	-	-	12
*Pergularia demia* (Forssk.) Chiov.	Lf	A	-	-	-	-	-	-	12	-
		M	11	12	-	-	-	-	13	11
*Hemidesmus indicus* (L.) R. Br. ex Schult.	Lf	A	-	-	-	-	-	-	-	-
		M	16	12	18	-	-	14	13	13
	St	A	-	-	-	-	-	-	12	-
		M	14	-	-	-	-	-	14	-
**Asteraceae**										
*Ageratum conyzoides* (L.) L.	Wp	A	-	11	12	12	11	-	12	-
		M	10	16	10	12	-	13	12	10
*Blumea lacera* (Burm.f.) DC.	Lf	A	-	-	-	-	-	-	-	-
		M	-	12	-	-	-	-	-	-
*Chrysanthellum americanum* (L.) Vatke	Lf	A	10	12	-	-	-	-	-	-
		M	11	13	-	-	-	-	-	-
*Elephantopus scaber* L.	Lf	A	14	10	-	-	11	-	08	-
		M	12	12	-	-	14	11	10	9
*Tridax procumbens* (L.) L.	Lf	A	-	-	-	-	-	-	-	13
		M	13	14	-	-	-	11	-	12
*Vernonia aspera* (Roxb.) Ham.	Lf	A	09	12	-	-	-	-	-	-
		M	11	14	-	-	-	-	-	-
*Vernonia squarrosa* Dinter ex Merxm.	Lf	A	-	-	-	-	12	-	-	10
		M	-	-	-	-	11	-	-	12
*Baccharoides anthelmintica* (L.) Moench	Lf	A	-	-	10	-	-	-	-	10
		M	-	-	14	-	-	-	-	12
**Bignoniaceae**										
*Oroxylum indicum* (L.) Kurz	Bk	A	12	10	-	-	-	12	12	-
		M	14	12	-	-	13	12	16	14
**Caesalpiniaceae**										
*Bauhinia variegata* L.	Lf	A	-	-	-	-	-	-	-	-
		M	11	10	-	-	-	-	-	-
*Cassia fistula* L.	Lf	A	13	12	10	09	11	12	08	12
		M	12	14	12	12	10	14	12	13
*Cassia occidentalis* L.	Lf	A	-	12	10	11	-	-	-	-
		M	-	11	12	-	-	-	-	-
*Cassia tora* L.	Lf	A	-	-	-	-	-	-	-	-
		M	-	12	12	-	12	-	-	-
*Saraca asoca* (Roxb.) Willd.	Lf	A	-	-	-	-	-	-	-	-
		M	-	10	-	-	-	-	-	-
*Tamarindus indica* L.	Lf	A	10	11	-	10	-	-	-	12
		M	12	10	08	12	-	-	-	14
***Calophyllaceae***										
*Mesua ferrea* L.	Lf	A	12	10	10	12	12	-	12	-
		M	12	10	10	10	12	-	12	-
**Capparaceae**										
*Capparis zeylanica* L.	Lf	A	-	-	-	-	-	-	-	-
		M	-	10	-	-	-	-	-	-
*Cleome viscosa* L.	Lf	A	10	11	-	-	10	-	-	-
		M	17	12	12	-	11	13	12	10
**Celastraceae**										
*Celastrus paniculatus* Willd.	Lf	A	-	12	-	-	-	13	-	15
		M	12	16	10	10	-	15	-	18
*Euonymus glaber* Roxb.	Lf	A	-	12	12	12	-	-	-	13
		M	12	20	14	16	-	12	-	16
**Clusiaceae**										
*Garcinia cowa* Roxb. ex Choisy	Lf	A	12	11	10	14	-	12	-	-
		M	12	13	10	12	10	10	12	10
**Cochlospermaceae**										
*Cochlospermum religiosum* (L.) Alston	Lf	A	-	-	-	-	-	-	-	
		M	-	10	-	-	-	-	-	
**Combretaceae**										
*Anogeissus latifolia* (Roxb. ex DC.) Wall. Ex Guillem. & Perr.	Lf	A	12	11	10	12	12	-	-	-
		M	14	08	11	12	12	10	-	11
*Combretum roxburghii* Spreng.	Lf	A	-	-	-	-	-	-	-	-
		M	-	22	-	-	12	14	-	16
*Terminalia alata* Heyne ex Roth	Bk	A	-	-	-	-	-	12	-	14
		M	14	12	-	-	-	11	12	12
*Terminalia arjuna* (Roxb. ex DC.) Wight & Arn.	Bk	A	-	12	-	12	14	11	11	12
		M	11	12	10	12	15	12	14	14
*Terminalia bellirica* (Gaertn.) Roxb.	Bk	A	10	12	11	13	-	-	10	-
		M	-	14	-	12	-	-	-	-
*Terminalia chebula* Retz.	Bk	A	-	-	-	-	-	-	-	-
		M	12	-	-	-	-	12	10	-
*Terminalia tomentosa* Wight & Arn.	Lf	A	-	-	-	-	-	-	-	10
		M	13	10	12	14	-	12	-	12
**Commelinaceae**										
*Commelina paludosa* Blume	Lf	A	14	-	12		-	-	-	-
		M	11	10	-	13	-	-	-	-
**Convolvulaceae**										
*Erycibe paniculata* Roxb.	Lf	A	-	10	-	-	-	-	-	10
		M	10	10	12	12	-	-	-	14
**Crassulaceae**										
*Kalanchoe pinnata* (Lam.) Pers.	Lf	A	-	-	-	-	-	-	-	-
		M	12	12	-	-	-	-	-	-
**Cucurbitaceae**										
*Coccinia grandis* (L.) Voigt	Lf	A	-	12	-	11	-	-	-	-
		M	12	11	-	12	-	12	-	-
*Momordica charantia* L.	Lf	A	10	-	-	-	-	-	-	-
		M	10	-	-	-	12	12	-	-
**Cyperaceae**										
*Cyperus rotundus* L.	Lf	A	11	10	-	10	-	-	-	-
		M	13	12	-	12	10	-	-	-
**Dilleniaceae**										
*Dillenia pentogyna* Roxb.	Lf	A	12	-	-	12	-	-	-	-
		M	10	12	-	12	12	-	10	-
**Dipterocarpaceae**										
*Shorea robusta* Gaertn.	Lf	A	10	-	-	-	-	12	-	11
		M	12	-	-	12	-	12	-	13
**Ebenaceae**										
*Diospyros malabarica* (Desr.) Kostel	Lf	A	-	-	-	-	-	-	-	-
		M	11	-	-	12	-	-	-	-
*Diospyros melanoxylon* Roxb.	Lf	A	-	-	10	11	-	-	12	-
		M	10	15	18	12	-	-	14	-
**	Bk	A	14	10	10	12	-	12	13	11
		M	15	11	22	16	-	10	16	10
*Diospyros montana* Roxb.	Lf	A	-	-	-	-	-	10	-	-
		M	12	-	-	-	-	10	-	10
*Diospyros sylvatica* Roxb.	Lf	A	-	12	-	-	-	-	-	-
		M	14	14	10	20	-	14	-	18
**Euphorbiaceae**										
*Antidesma ghaesembilla* Gaertn.	Lf	A	-	-	-	-	-	-	-	-
		M	13	12	-	-	-	-	-	-
*Cleistanthus collinus* (Roxb.) Benth ex Hook. f.	Lf	A	12	10	12	-	-	12	-	12
		M	10	12	14	14	-	12	10	12
*Croton caudatus* Geiseler	Lf	A	-	-	-	-	-	-	-	-
		M	10	-	-	-	-	-	-	-
*Croton roxburghii* Wall.	Lf	A	10	16	10	-	12	12	-	13
		M	12	14	17	15	15	13	12	10
*Croton roxburghii* Wall.	Bk	A	-	12	15	14	-	-	14	-
		M	12	14	20	15	-	-	17	-
*Emblica officinalis* Gaertn.	Lf	A	-	12	10	10	-	-	-	-
		M	11	10	12	-	12	-	-	-
*Euphorbia hirta* L.	Lf	A	-	10	12	-	10	12	-	-
		M	10	-	14	-	12	10	-	-
*Jatropha gossypiifolia* L.	Lf	A	-	-	-	-	-	-	-	-
		M	-	-	10	12	-	-	-	-
*Macaranga peltata* (Roxb.) Mull. Arg.	Lf	A	-	-	-	-	-	-	-	-
		M	-	-	10	-	-	-	-	-
*Mallotus philippensis* (Lam.) Mull. Arg.	Lf	A	-	-	-	-	-	-	-	-
		M	12	14	-	-	-	-	-	-
*Phyllanthus fraternus* G. L. Webster	Wp	A	-	-	-	-	-	-	-	10
		M	-	-	-	-	-	-	-	-
*Ricinus communis* L.	Lf	A	12	-	-	-	-	10	10	-
		M	10	14	-	12	10	12	12	10
**Flacourtiaceae**										
*Flacourtia jangomas* (Lour.) Raeusch.	Lf	A	-	12	10	-	-	12	-	11
		M	-	12	12	-	-	-	-	-
**Fabaceae**										
*Butea monsperma* (Lam.) Taub.	Lf	A	-	10	-	-	-	-	-	-
		M	12	10	-	-	-	-	-	-
*Butea superba* Roxb.	Lf	A	-	10	10	10	-	-	-	-
		M	12	10	10	-	-	-	-	-
*Clitoria ternatea* L.	Lf	A	-	-	-	-	-	-	-	-
		M	-	10	-	-	-	-	-	-
*Dalbergia latifolia* Roxb.	Bk	A	-	-	-	-	-	-	-	-
		M	-	12	12	-	-	-	-	-
*Dalbergia volubilis* Roxb.	Bk	A	-	-	-	-	-	-	-	-
		M	-	12	-	-	-	-	-	-
*Desmodium gangeticum* (L.) DC.	Lf	A	12	-	08	-	10	-	-	-
		M	10	12	10	10	12	-	-	-
*Desmodium oojeinense* (Roxb.) H. Ohashi	Lf	A	-	-	-	-	-	-	-	-
		M	-	10	-	-	-	-	-	-
*Desmodium pulchellum* (L.) Benth.	Lf	A	-	-	10	-	-	-	-	-
		M	-	12	12	-	-	-	-	-
*Flemingia nana* Roxb.	Rt	A	15	11	-	-	-	12	-	10
		M	14	12	10	10	-	12	-	12
*Glycyrrhiza glabra* (L.)	Bk	A	-	11	-	-	-	-	-	10
		M	12	10	-	-	-	-	-	18
*Indigofera cassioides* DC.	Lf	A	-	-	-	-	-	-	-	-
		M	14	12	-	10	-	-	-	10
*Indigofera glabra* L.	Lf	A	-	11	-	09	-	-	-	-
		M	-	-	-	-	-	-	-	-
*Millettia extensa* (Benth) Baker	Lf	A	-	12	-	-	-	-	-	-
		M	11	14	20	-	10	11	-	-
*Pterocarpus marsupium* Roxb.	Bk	A	-	12	-	10	-	10	-	-
		M	12	-	-	12	-	14	-	-
*Tephrosia purpurea* (L.) Pers.	Fr	A	-	-	-	12	-	-	-	-
		M	-	-	-	10	-	-	-	-
**Gentianaceae**										
*Canscora decurrens* Daizell	Wp	A	-	-	-	-	-	-	-	-
		M	-	12	09	-	-	-	-	-
**Iridaceae**										
*Eleutherine bulbosa* (Mill.) Urb.	Bl	A	18	16	10	17	-	12	-	-
		M	25	18	14	15	11	17	-	-
**Lamiaceae**										
*Hyptis suaveolens* (L.) Poit.	Lf	A	12	-	-	-	-	-	-	-
		M	14	-	-	-	-	-	-	-
*Ocimum americanum* L.	Lf	A	-	09	-	-	10	-	-	-
		M	-	10	-	-	12	-	-	12
*Ocimum sanctum* L.	Lf	A	-	-	-	10	10	-	-	-
		M	-	12	-	10	10	-	10	-
**Lauraceae**										
*Litsea glutinosa* (Lour.) C.B. Rob.	Lf	A	-	-	-	-	-	-	-	-
		M	-	-	10	11	-	-	-	-
**Leguminosae**										
*Abrus precatorius* L.	Lf	A	-	-	-	-	-	-	-	-
		M	-	12	-	-	-	-	-	-
	Fr	A	-	-	-	-	-	-	-	-
		M	-	-	-	-	-	-	12	-
**Linaceae**										
*Linum usitatissimum* L.	Lf	A	-	-	-	-	-	-	-	-
		M	-	10	-	-	-	-	-	-
**Loranthaceae**										
*Dendrophthoe falcata* (L.f.) Ettingsh.	Lf	A	-	-	-	-	-	-	-	-
		M	-	10	-	-	-	-	-	-
**Lythraceae**										
*Lagerstroemia speciosa* (L.) Pers.	Lf	A	-	-	10	12	-	-	-	-
		M	-	12	12	-	-	-	-	-
**Malvaceae**										
*Sida acuta* Burm. f.	Lf	A	-	10	-	-	-	-	-	-
		M	-	14	14	-	-	-	-	-
*Sida cordata* (Burm.f.) Borss.Waalk.	Wp	A	-	-	-	-	-	-	-	-
		M	12	10	-	-	-	-	-	-
****Marattiaceae****										
*Angiopteris evecta* (G. Forst.) Hoffm.	Lf	A	-	-	-	-	-	-	-	-
		M	-	12	-	-	-	14	-	13
**Melastomataceae**										
*Melastoma malabathricum* L.	Bk	A	-	10	-	-	-	-	-	10
		M	-	16	-	-	16	-	-	20
**Meliaceae**										
*Azadirachta indica* A. Juss.	Bk	A	15	-	10	-	-	10	-	-
		M	16	11	12	-	12	15	-	12
**Menispermaceae**										
*Cissampelos pareira* L.	Rt	A	-	-	-	12	-	10	-	-
		M	12	12	12	14	10	12	-	10
**Mimosoideae**										
*Acacia leucophloea* (Roxb.) Willd.	Lf	A	-	-	-	09	-	-	-	-
		M	14	-	10	14	12	-	-	10
**Moraceae**										
*Ficus racemosa* L.	Bk	A	-	-	12	-	-	-	-	-
		M	16	-	14	12	-	-	10	14
**Moringaceae**										
*Moringa oleafera* Lam.	Lf	A	-	19	18	-	-	15	-	08
		M	11	16	12	12	10	14	12	12
**Myrsinaceae**										
*Ardisia solanacea* (Poir.) Roxb.	Lf	A	-	10	10	-	-	-	-	-
		M	10	12	12	10	-	14	-	-
**Myrtaceae**										
*Eucalyptus citriodora* Hook.	Bk	A	-	-	-	-	-	-	-	-
		M	-	-	-	-	11	10	-	-
*Psidium guajava* L.	Lf	A	-	11	-	12	-	-	-	-
		M	-	12	-	14	-	-	-	-
*Syzygium cumini* (L.) Skeels	Lf	A	-	10	-	-	09	-	-	10
		M	14	11	-	-	12	-	-	11
*Syzygium jambos* (L.) Alston	Lf	A	-	12	-	-	-	-	-	10
		M	-	10	-	-	-	-	10	12
**Oleaceae**										
*Nyctanthes arbor-tristis* L.	Lf	A	-	14	10	12	-	-	10	10
		M	20	22	15	10	-	-	18	13
	Bk	A	10	10	10	14	-	-	10	10
		M	22	14	22	11		-	15	18
**Onagraceae**										
*Ludwigia octovalvis* (Jacq.) P.H. Raven	Lf	A	-	09	-	-	-	-	-	-
		M	-	12	-	-	-	-	-	-
**Papaveraceae**										
*Argemone mexicana* L.	Lf	A	-	-	-	-	-	-	-	-
		M	-	-	-	-	-	-	-	12
**Peripiocaceae**										
*Hemidesmus indicus* (L.) R.Br. ex Schult.	Lf	A	11	-	12	-	10	-	-	-
		M	12	10	13	-	10	-	-	-
**Polypodiaceae**										
*Drynaria quercifolia* (L.) J. Sm.	St	A	-	-	-	-	-	-	-	-
		M	12	15	-	-	-	-	-	-
**Punicaceae**										
*Punica granatum* L.	Lf	A	10	12	10	12	-	12	-	14
		M	17	12	-	10	-	10	-	12
**Rhamnaceae**										
*Ziziphus mauritiana* Lam.	Lf	A	-	10	-	-	-	-	-	-
		M	-	12	-	-	10	-	-	-
**Rubiaceae**										
*Anthocephalus chinensis* (Lam.) Hassk.	Lf	A	-	10	12	-	-	-	-	-
		M	-	12	12	-	10	-	-	-
*Canthium dicoccum* (Gaertn.) Merr.	Lf	A	10	-	-	-	-	-	-	-
		M	14	-	-	-	-	-	-	-
*Ixora pavetta * Andr.	Lf	A	10	-	-	-	-	-	-	-
		M	10	10	-	-	-	10	-	-
*Mitragyna parvifolia* (Roxb.) Korth.	Lf	A	-	-	-	-	-	-	-	08
		M	11	08	-	-	-	10	-	12
*Paederia foetida * L.	Lf	A	-	-	-	-	-	-	-	08
		M	12	12	-	-	-	12	-	12
*Wendlandia tinctoria* (Roxb.) DC	Lf	A	12	-	-	10	-	10	-	-
		M	-	-	-	10	10	12	-	-
**Rutaceae**										
*Acronychia pedunculata * (L.) Miq.	Lf	A	-	10	-	-	-	-	-	-
		M	-	12	-	-	12	-	-	-
*Aegle marmelos* (L.) Correa	Lf	A	-	10	-	-	-	-	-	-
		M	-	12	-	-	-	12	-	10
*Citrus aurantium* L.	Lf	A	-	-	-	-	-	-	-	-
		M	10	12	-	-	-	-	-	-
*Clausena excavate * Burm. f.	Lf	A	11	09	-	14	-	-	-	-
		M	13	12	14	12	-	-	-	12
*Murraya koenigii* (L.) Spreng.	Lf	A	12	10	-	-	-	12	-	-
		M	12	-	-	-	-	10	-	-
**Sapindaceae**										
*Schleichera oleosa* (Lour.) Merr.	Lf	A	-	10	-	-	-	12	-	-
		M	-	-	-	-	-	10	-	-
**Sapotaceae**										
*Madhuca longifolia * (J.Koenig ex L.) J.F.Macbr.	Lf	A	12	10	-	-	-	12	-	-
		M	-	-	-	-	-	10	-	-
*Mimusops elengi* L.	Lf	A	-	10	-	-	-	12	-	-
		M	11	-	-	-	-	10	-	-
**Scrophulariaceae**										
*Scoparia dulcis* L.	Lf	A	12	10	-	-	09	-	-	-
		M	14	12	-	10	11	-	-	-
**Solanaceae**										
*Datura metel* L.	Lf	A	-	-	-	-	-	-	-	-
		M	-	-	-	12	10	-	-	-
*Solanum virginianum* L.	Lf	A	-	-	-	10	-	-	-	-
		M	-	10	-	11	-	-	-	-
**Sterculiaceae**										
*Helicteres isora* L.	Lf	A	-	-	-	12	-	-	-	-
		M	11	10	-	10	-	-	-	-
	Rt	A	-	-	-	10	-	-	-	-
		M	12	11	12	12	-	-	12	13
*Pterospermum acerifolium* (L.) Willd.	Lf	A	-	-	-	12	-	-	12	-
		M	15	11	10	15	-	14	10	-
*Pterospermum xylocarpum * (Gaertn.) Sant. & Wagh	Lf	A	-	-	-	-	-	-	-	-
		M	-	12	-	-	-	-	-	-
**Tilliaceae**										
*Grewia elastica * Royle	Lf	A	-	-	-	-	-	-	-	-
		M	-	-	-	-	13	-	10	-
**Ulmaceae**										
*Trema orientalis* (L.) Blume	Lf	A	-	10	-	-	-	-	-	-
		M	15	12	-	-	-	-	-	-
**Verbenaceae**										
*Clerodendrum indicum* (L.) Kuntze	Lf	A	-	10	10	-	14	-	10	-
		M	12	14	12	-	12	11	10	09
*Clerodendrum viscosum * Vent.	Lf	A	14	-	-	-	-	-	-	-
		M	13	-	-	-	-	-	10	-
*Lantana camara* L.	Lf	A	-	-	12	-	-	-	-	-
		M	-	-	-	-	-	-	10	-
*Vitex negundo * L.	Lf	A	10	12	10	-	-	-	-	-
									
		M	18	16	12	10	-	-	18	14
	Bk	A	12	12	10	12	-	-	10	10
		M	14	13	18	17	-	-	12	16
**Vitaceae**										
*Leea indica * (Burm. f.) Merr.	Lf	A	-	-	-	-	-	-	-	-
		M	-	-	12	-	-	10	-	-
*Cissus quadrangularis * L.	Wp	A	-	-	-	-	-	-	-	-
		M	-	-	10	-	-	-	-	10
**Zingiberaceae**										
*Curcuma anguistifolia* Roxb.	Lf	A	-	-	-	-	-	10	-	-
		M	-	-	-	10	-	08	-	08
*Curcuma aromatic * Salisb.	Rh	A	-	-	-	-	-	-	-	-
		M	11	-	-	-	-	-	-	12
*Kaempferia rotunda* L.	Lf	A	-	-	-	-	-	-	-	-
		M	13	-	-	-	-	-	11	-
Antibiotic-Ciprofloxacin			22	16	16	24	20	26	23	R
Antibiotic-Gentamicin			27	24	26	18	22	24	21	20

PU. Parts used; E. Extract; A. Aqueous; M. Methanol; Fl. flower; Fr. fruit; Lf. leaf; Bk. bark; Rt. root; Rh. rhizome; St. stem; Sd. seeds; Wp. whole plant; Bacterial species: Bc. *B. cereus*; Sa. *S. aureus*; Ec. *E. coli*; St. *S. typhimurium*; Sd. *S. dysentriae*; Sf. *S. flexneri*; Ss. *S. sonnei*; Vc. *V. cholera*.

**Table 3:** Summary of antibacterial activity among the test plants.

Scrutiny	No. of Extracts Reported as Antibacterial (%)		
	Organism	Methanol Extract	Aqueous Extract
Total number of plant species tested—222	Gram positive	146 (56.58%)	89 (34.49%)
Total number of Genus tested—177	Gram negative	137 (53.10%)	102 (39.53%)
Total number of family tested—83	*B. cereus*	108 (41.86%)	50 (19.37%)
Total number of parts tested = 258	*S. aureus*	124 (48.06%)	76 (29.45%)
Leaves-125; Bark-19; Whole part-08; Stem-04	*E. coli*	68 (26.35%)	45 (17.44%)
Root-04; Rhizome-03; Fruit-03 and Bulb-01	*S. typhimurium*	65 (25.19%)	41 (15.89%)
Total number of methanol extracts active—165	*S. dysentriae*	50 (19.37%)	22 (8.52%)
Total number of aqueous extracts active—127	*S. flexneri*	66 (25.58%)	28 (10.85%)
Number of species do not show activity-90 species	*S. sonnei*	47 (18.21%)	24 (9.30%)
Number of extracts do not show activity	*V. cholerae*	72 (27.90%)	38 (14.72%)
(93 methanol + 131 aqueous = 224)	Zone ≥ 20 mm	10 (3.87%)	0
Total number of family show activity—68	Zone (15–20) mm	34 (13.17%)	9 (3.48%)
Total number of family do not show activity—15	Zone < 15	160 (62.01%)	121 (46.89%)

**Table 4:** Minimum inhibitory concentration (MIC) results of selected plants from SBR.

Plant Species	Plant Part	Test Bacteria			
		Sa	Bc	Sf	Vc
*Achyranthes aspera*	Rt	>4000	>4000	>4000	2000
*Acorus calamus*	Rh	>5000	>5000	>5000	>5000
*Adhatoda vasica *	Lf	500	500	1000	2000
*Aegle marmelos*	Lf	>4000	>4000	4000	>4000
*Ageratum conyzoides*	Wp	500	>4000	500	4000
*Alangium salvifolium*	Lf	>5000	>5000	>5000	>5000
*Alpinia galanga*	Lf	1000	1000	2000	500
*Alstonia scholaris*	Lf	>2000	>2000	1000	500
*Andrographis paniculata*	Lf	1000	1000	2000	500
*A. paniculata*	St	500	1000	2000	1000
*Angiopteris evecta*	Lf	>4000	>4000	2000	>5000
*Anogeissus latifolia*	Lf	1000	4000	1000	1000
*Annona squamosa*	Lf	1000	2000	1000	1000
*Annona reticulata*	Lf	1000	2000	1000	1000
*Ardisia solanacea*	Lf	1000	2000	1000	4000
*Azadirachta indica*	Lf	250	250	250	250
*Buchanania lanzan*	Bk	187	312	625	625
*Cassia fistula*	Lf	94	312	625	625
*Celastrus paniculatus*	Lf	1000	500	1000	500
*Centella asiatica*	Wp	1000	1000	1000	2000
*Cissampelos pareira*	Lf	>4000	500	500	1000
*Clausena excavata*	Lf	1250	625	1250	1250
*Cleome viscosa*	Lf	1000	500	500	1000
*Cleistanthus collinus*	Lf	1250	1250	1250	2500
*Clerodendrum indicum*	Lf	250	2000	250	500
*Combretum roxburghii*	Bk	1250	1250	2500	2500
*Croton roxburghii*	Lf	625	625	625	156
*C. roxburghii*	Bk	312	312	>5000	5000
*Diospyros melanoxylon*	Lf	>5000	>5000	>5000	2500
*D. melanoxylon*	Bk	1000	250	500	250
*Diospyros sylvatica *	Bk	1250	625	625	1250
*Elephantopus scaber*	Lf	2000	250	2000	250
*Eleutherine bulbosa*	Bl	62	22	125	125
*Erycibe paniculata*	Lf	500	500	1250	1250
*Eryngium foetidum*	Lf	2500	2500	2500	2500
*E. foetidum*	St	1250	1250	5000	1250
*Euonymus glaber*	Lf	250	500	1000	2000
*Flemingia nana*	Rt	4000	1000	>4000	4000
*Garcinia cowa*	Lf	625	1250	1250	1250
*Helicteres isora*	Rt	1250	1250	1250	1250
*Hemidesmus indicus*	Lf	4000	1000	4000	4000
*Holarrhena antidysenterica*	Lf	1250	312	625	2500
*Lannea coromandelica*	Lf	625	312	2500	2500
*Millettia extensa*	Lf	2500	>5000	>5000	>5000
*Mimusops elengi*	Lf	5000	>5000	2500	>5000
*Momordica dioica*	Lf	>5000	>5000	>5000	>5000
*Mimusops elengi *	Lf	1000	4000	2000	4000
*Moringa oleafera*	Lf	625	312	2500	2500
*Nyctanthes arbor-tristis*	Lf	312	312	1250	312
*N. arbor-tristis*	Bk	156	156	156	625
*Oroxylum indicum*	Bk	250	250	500	125
*Paederia foetida*	Lf	1000	1000	2000	1000
*Pterospermum acerifolium*	Bk	312	312	1250	>5000
*Punica granatum*	Lf	625	1250	2500	2500
*Ricinus communis*	Lf	1000	1000	>5000	1000
*Semecarpus anacardium*	Fr	500	2000	500	2000
*Shorea robusta*	Lf	4000	2000	>4000	>4000
*Spondias pinnata*	Lf	500	500	500	500
*Tamarindus indica*	Lf	2000	2000	>4000	>4000
*Terminalia alata*	Bk	625	312	2500	2500
*Terminalia arjuna*	Bk	1000	2000	>4000	4000
*Terminalia tomentosa*	Bk	2500	2500	2500	2500
*Tridax procumbens*	Lf	3000	>6000	>6000	>6000
*Vitex negundo*	Lf	>5000	2500	1250	5000
*V. negundo*	Bk	>5000	>5000	>5000	>5000

A. aqueous; M. methanol; Fl. flower; Fr. fruit; Lf. leaf; Bk. bark; Rt. root; Rh. rhizome; Bl. bulb; St. stem; Sd. seeds; Wp. whole plant; Sa. *S. aureus;* Bs. *B. cereus;* Sf. *S. flexneri;* and Vc. *V. cholerae*. MIC values are expressed in µg/mL. The stock extracts concentrations were 20 mg/mL; 25 mg/mL and 30 mg/mL.

**Table 5:** Antibiogram among the test bacterial strains.

Antibiotic(s)	Bacterial Strains (Zone of Inhibition in mm)							
	Bs	Sa	Ec	St	Sd	Sf	Ss	Vc
Amikacin	R	R	R	R	R	R	R	R
Ampicillin	R	18	R	R	12	14	R	R
Ciprofloxacin	22	16	16	24	20	26	23	R
Erythromycin	20	23	R	R	R	R	16	18
Gatifloxacin	22	22	18	19	14	18	R	R
Gentamicin	27	24	26	18	22	24	21	20
Vancomycin	20	16	19	15	14	17	23	14
Streptomycin	18	26	22	14	18	14	25	R
Tetracycline	22	14	23	18	14	13	17	16
Amoxicillin	14	R	R	R	R	12	14	R
Cefoxitin	R	R	R	R	R	15	26	21
Cephotaxime	R	R	14	R	26	22	20	17
Ceftriaxone	14	17	16	18	22	28	32	18
Ofloxacin	23	21	18	19	14	23	24	15
Levofloxacin	19	22	2R	18	18	2R	18	16
Chloramphencol	17	19	29	23	R	14	12	R
Nalidaxic acid	R	R	R	R	25	28	R	R
Polymyxin B	14	R	12	R	14	12	R	R

R—Resistant; Bc. *B. cereus*; Sa. *S. aureus*; Ec. *E. coli*; St. *S. typhimurium*; Sd. *S. dysentriae*; Sf. *S. flexneri*; Ss. *S. sonnei*; Vc. *V. cholerae*.

## Abbreviations

The following abbreviations are used in this manuscript:

CFU: Colony forming unit
MDR: Multiple drug resistance
MHA: Muller-Hinton agar
MTCC: Microbial type culture collection
NA: Nutrient agar

## References

[1] Mohanta T.K., Occhipinti A., Atsbaha Zebelo S., Foti M., Fliegmann J., Bossi S., Maffei M.E., Bertea C.M. (2012). Ginkgo biloba responds to herbivory by activating early signaling and direct defenses. PLoS ONE.

[2] Mohanta T.K., Tamboli Y., Zubaidha P.K. (2014). Phytochemical and medicinal importance of *Ginkgo biloba* L.. Nat. Prod. Res..

[3] Verma S., Singh S.P. (2008). Current and future status of herbal medicines. Vet. World.

[4] Dubey N.K., Kumar R., Tripathi P. (2004). Global promotion of herbal medicine: India’s opportunity. Curr. Sci..

[5] Cown M.M. (1999). Plant products as antimicrobial agents. Clin. Microbiol. Rev..

[6] Parekh J., Chanda S.V. (2008). *In vitro* antimicrobial activity and phytochemical analysis of some Indian medicinal plants. Turk. J. Biotechnol..

[7] Fabry W., Okemo P.O., Ansorg R. (1998). Antibacterial activity of East African medicinal plants. J. Ethnopharmacol..

[8] Vlietinck A.J., Vanden Berghe D.A. (1991). Can ethnopharmacology contribute to the development of antiviral drugs?. J. Ethnopharmacol..

[9] Fabricant D.S., Farnsworth N.R. (2001). The Value of Plants Used in Traditional Medicine for Drug Discovery. Environ. Heal..

[10] Vuorela P., Leinonen M., Saikku P., Tammela P., Rauha P., Wennberg T., Vuorela H. (2004). Natural Products in the Process of Finding New Drug Candidates. Curr. Med. Chem..

[11] Eloff J.N., Katerere D.R., McGaw L.J. (2008). The biological activity and chemistry of the southern African Combretaceae. J. Ethnopharmacol..

[12] Pauw E., Eloff J. (2014). Which tree orders in southern Africa have the highest antimicrobial activity and selectivity against bacterial and fungal pathogens of animals?. BMC Complement. Altern. Med..

[13] Lewis K., Ausubel F.M. (2006). Prospects for plant-derived antibacterials. Nat. Biotech..

[14] Panda S.K. (2014). Ethno-medicinal uses and screening of plants for antibacterial activity from Similipal Biosphere Reserve, Odisha, India. J. Ethnopharmacol..

[15] Padhi L., Panda S. (2015). Antibacterial activity of Eleutherine bulbosa (Miller) Urban (Iridaceae) against multidrug resistant bacteria. J. Acute Med..

[16] Silva G., Lee I., Kinghor A., Cannel R. (1998). Special problems with the extraction of plants. Methods in Biotechnology.

[17] Panda S.K., Niranjan P., Gunanidhi S., Bastia A.K., Dutta S.K. (2012). Anti-diarrheal activities of medicinal plants of Similipal Biosphere Reserve, Odisha, India. Int. J. Med. Aromat. Plants.

[18] Lin J., Opoku A.R., Geheeb-Keller M., Hutchings A.D., Terblanche S.E., Jäger A.K., Van Staden J. (1999). Preliminary screening of some traditional zulu medicinal plants for anti-inflammatory and anti-microbial activities. J. Ethnopharmacol..

[19] Romero C.D., Chopin S.F., Buck G., Martinez E., Garcia M., Bixby L. (2005). Antibacterial properties of common herbal remedies of the southwest. J. Ethnopharmacol..

[20] Nikaido H., Neidhardt F.C. (1996). Outer membrane In *Escherichia coli* and Salmonella. Cellular and Molecular Biology.

[21] Yerra R., Gupta M., Mazumder U. (2005). *In Vitro* Lipid Peroxidation and Antimicrobial Activity of Mucuna pruriens Seeds. Iran. J. Pharmacol. Ther..

[22] Kuete V., Nguemeving J.R., Beng V.P., Azebaze A.G.B., Etoa F.-X., Meyer M., Bodo B., Nkengfack A.E. (2007). Antimicrobial activity of the methanolic extracts and compounds from Vismia laurentii De Wild (Guttiferae). J. Ethnopharmacol..

[23] Ahmad I., Mehmood Z., Mohammad F. (1998). Screening of some Indian medicinal plants for their antimicrobial properties. J. Ethnopharmacol..

[24] Valsaraj R., Pushpangadan P., Smitt U.W., Adsersen A., Nyman U. (1997). Antimicrobial screening of selected medicinal plants from India. J. Ethnopharmacol..

[25] Samy R.P., Ignacimuthu S. (2000). Antibacterial activity of some folklore medicinal plants used by tribals in Western Ghats of India. J. Ethnopharmacol..

[26] Srinivasan D., Nathan S., Suresh T., Lakshmana Perumalsamy P. (2001). Antimicrobial activity of certain Indian medicinal plants used in folkloric medicine. J. Ethnopharmacol..

[27] Ahmad I., Beg A.Z. (2001). Antimicrobial and phytochemical studies on 45 Indian medicinal plants against multi-drug resistant human pathogens. J. Ethnopharmacol..

[28] Jeevan Ram A., Bhakshu L.M., Venkata Raju R.R. (2004). In vitro antimicrobial activity of certain medicinal plants from Eastern Ghats, India, used for skin diseases. J. Ethnopharmacol..

[29] Kumar V.P., Chauhan N.S., Padh H., Rajani M. (2006). Search for antibacterial and antifungal agents from selected Indian medicinal plants. J. Ethnopharmacol..

[30] Parekh J., Chanda S.V. (2008). Antibacterial activity of aqueous and alcoholic extracts of 34 Indian medicinal plants against some Staphylococcus species. Turk. J. Biol..

[31] Ahmad I., Aqil F. (2007). *In vitro* efficacy of bioactive extracts of 15 medicinal plants against ESβL-producing multidrug-resistant enteric bacteria. Microbiol. Res..

[32] Posadzki P., Watson L.K., Ernst E. (2013). Adverse effects of herbal medicines: An overview of systematic reviews. Clin. Med. J. R. Coll. Phys. Lond..

[33] Panda S. (2015). Methods to study antimicrobial and antioxidant properties of medicinal plants. Advances in Natural Products.

[34] Mohanta T., Patra J., Rath S. (2007). Evaluation of antimicrobial activity and phytochemical screening of oils and nuts of Semicarpus anacardium. Sci. Res. Essay.

[35] Bauer A.W., Kirby W.M., Sherris J.C., Turck M. (1966). Antibiotic susceptibility testing by a standardized single disk method. Am. J. Clin. Pathol..

