# Erratum: Panda, S.K., et al., Large-Scale Screening of Ethnomedicinal Plants for Identification of Potential Antibacterial Compounds. *Molecules* 2016, *21*, 293

**DOI:** 10.3390/molecules25081897

**Published:** 2020-04-20

**Authors:** Sujogya Kumar Panda, Yugal Kishore Mohanta, Laxmipriya Padhi, Young-Hwan Park, Tapan Kumar Mohanta, Hanhong Bae

**Affiliations:** 1 Department of Zoology, North Orissa University, Baripada, Odisha 757003, India; sujogyapanda@gmail.com (S.K.P.); laxmipriyapadhi85@gmail.com (L.P.); 2 Department of Botany, North Orissa University, Baripada, Odisha 757003, India; ykmohanta@gmail.com; 3 School of Biotechnology, Yeungnam University, Gyeongsan 712749, Korea; pyhasdf@nate.com; 4 Free Major of Natural Sciences, College of Basic Studies, Yeungnam University, Gyeongsan 712749, Korea

The authors wish to make the following corrections to their paper [1]. The authors regret that in the above-mentioned paper, page 4, Table 2, under sub-heading family Araceae, *Acorus calamus* L. should be changed to *Colocasia esculenta* (L.) Schott. The authors would like to apologize for any inconvenience caused to the readers by these changes.

## Figures and Tables

**Table 2:** Results of screening of plants from Northern Odisha, India.

Plant Description	Zone of Inhibition in mm									
Araceae	PU	E	Bc	Sa	Ec	St	Sd	Sf	Ss	Vc
*Colocasia esculenta* (L.) Schott	Rh	A	-	-	09	-	12	-	-	09
		M	-	-	12	12	14	-	-	12
